# Sociodemographic aspects, beliefs about lifestyles, and religiosity as predictors of life satisfaction in Peruvian university students: a cross-sectional study

**DOI:** 10.3389/fpubh.2024.1476544

**Published:** 2024-10-14

**Authors:** Jacksaint Saintila, David Javier-Aliaga, Ana Valle-Chafloque, Christian Casas-Gálvez, Luz Antonia Barreto-Espinoza, Yaquelin E. Calizaya-Milla

**Affiliations:** ^1^Research Group for Nutrition and Healthy Behaviors, School of Medicine, Universidad Señor de Sipán, Chiclayo, Peru; ^2^Research Group for Nutrition and Lifestyle, Universidad Peruana Unión, Lima, Peru

**Keywords:** healthy lifestyle, personal satisfaction, religion, students university, sociodemographic characteristics, subjective well-being, Peru

## Abstract

**Introduction:**

Beliefs and practices related to healthy lifestyles and religiosity can play an important role in overall well-being. However, few studies have explored the association between these factors and life satisfaction in the university context. This study examined the association between sociodemographic aspects, lifestyle beliefs, and religiosity with life satisfaction in university students.

**Methods:**

A cross-sectional predictive study was conducted during the months of March and May 2024 at a private university located in the northern region of Peru. Validated instruments were distributed that included a lifestyle beliefs scale, the Santa Clara Strength of Religious Faith Questionnaire-Short Form (SCSRFQ-SF), the Satisfaction with Life Scale (SWLS) and a sociodemographic data section. A total of 1,258 students participated in the study. Multiple linear regression models were used for predictive analysis.

**Results:**

Being a student of foreign origin, compared to being Peruvian, is negatively and significantly associated with life satisfaction (*β* = −0.066, *p* = 0.003). In contrast, a positive association is observed between belonging to the Faculty of Health Sciences (*β* = 0.119, *p* = 0.048) and life satisfaction, compared to students from other faculties. Furthermore, lifestyle beliefs (*β* = 0.579; *p* < 0.001) and religiosity (*β* = 0.182; *p* < 0.001) are positively and significantly associated with life satisfaction.

**Conclusion:**

These findings suggest that universities and health professionals should consider promoting healthy lifestyles and supporting religious practices as important strategies to improve student life satisfaction, considering relevant sociodemographic aspects.

## 1 Introduction

In Peru, university life is characterized by distinct social and demographic features that have evolved over the past few decades (1). According to recent data, Peru saw significant growth in university enrollment between 2012 and 2021, with 45.3% of the population aged 19–23 attending higher education (2). This data indicates increased accessibility to higher education compared to 12 years ago, when university enrollment was both lower and less diverse (3). Today’s Peruvian students come from increasingly diverse socioeconomic and cultural backgrounds, transforming the demographic composition of universities (1). Furthermore, the social life of university students in Peru is shaped by the dynamics of a country characterized by high cultural diversity and a deep-rooted religious tradition (4).

On the other hand, university life is an important period of transition, marked by a series of challenges and profound changes that affect various dimensions of young people’s existence (5). For example, university students are experiencing lifestyle changes due to increased independence and time constraints (6). Furthermore, they face challenges related to academic pressure, making important academic decisions, and adapting to a new social environment, which can have a significant impact on physical, mental, and emotional health, leading to higher levels of academic stress and anxiety (7). Loneliness and isolation are also common problems, especially for those moving away from home (5). These conditions, along with anxiety about the future, and the need to balance academic and social life, can negatively affect the quality of life and satisfaction of students (6, 8). As students navigate these challenges, their awareness and beliefs about their health, as well as their ability to manage these changes, become crucial determinants of their overall well-being during this critical stage. Likewise, various sociodemographic factors such as age, provenance, residence, faculty affiliation, marital status, and body mass index (BMI) also play significant roles in shaping students’ life satisfaction.

In particular, beliefs about a healthy lifestyle—such as the importance of maintaining a balanced diet, engaging in regular physical activity, and effectively managing stress—can significantly influence how students cope with the demands of university life (9). These beliefs are not formed in isolation; they are often shaped by broader cultural, social, and even spiritual influences that provide a framework for how individuals perceive their health and well-being. One such influence is religiosity, which encompasses a person’s spiritual practices and beliefs. Religiosity can play a significant role in providing emotional support, fostering a sense of community, and offering a moral framework that helps students navigate the complexities of university life (10). The intersection of these beliefs and practices—both in terms of healthy lifestyle choices and religiosity—may therefore have a profound impact on a student’s life satisfaction, which is a key indicator of overall well-being.

### 1.1 Life satisfaction

Life satisfaction is conceptualized as a subjective assessment of an individual’s overall quality of life according to their own chosen criteria (11). It reflects a cognitive judgment process, wherein individuals compare their life circumstances to their own standards and expectations (11). Life satisfaction is a key component of subjective well-being, which encompasses not only the presence of positive emotions and the absence of negative emotions but also the satisfaction with life domains such as family, work, health, and social relationships (12). The Satisfaction with Life Scale (SWLS), used in this study, captures this global evaluation of life satisfaction through a series of statements that participants rate based on how much they agree or disagree with them (13). Thus, in the context of this research, life satisfaction serves as an essential indicator of the overall well-being of university students, shaped by their beliefs about healthy lifestyles and religiosity.

Empirical studies have shown that the level of life satisfaction among university students can vary widely, often classified into low, moderate, and high levels. Students with low life satisfaction are more likely to experience adverse mental health outcomes, such as depression, anxiety, and increased stress levels, which can negatively impact their academic performance and social relationships (14, 15). In contrast, students with high life satisfaction tend to exhibit better academic performance, stronger social relationships, and greater resilience to stress (16, 17). High levels of life satisfaction are also linked to positive health behaviors, such as regular physical activity and healthy eating, which further contribute to their overall well-being (18). Understanding these varying levels of life satisfaction and their potential consequences is important for developing targeted interventions that support students’ well-being. Universities that focus on enhancing life satisfaction among students may help mitigate the risks associated with low life satisfaction and promote a healthier, more productive academic environment.

### 1.2 Beliefs about healthy lifestyles

The term “beliefs about a healthy lifestyle” is used to describe a person’s conviction in their ability to adopt and maintain habits that promote healthy living (9). These beliefs include the perceived importance of a balanced diet, regular physical activity, taking responsibility for one’s health, managing stress, and engaging in behaviors that promote physical and mental health (19). Lifestyle beliefs formed at university will have a significant impact on all aspects of student life, including increased life satisfaction (7). Students who believe in the importance of a healthy lifestyle and implement it tend to have better academic performance and fewer health problems (20), which can increase overall satisfaction. Therefore, it is critical that universities actively promote these healthy habits and beliefs among students to support their holistic development and long-term life satisfaction.

The health belief model as a theoretical framework is one of the most widely used to explain both change and maintenance of style-related beliefs and behaviors in individuals and focusses on helping people understand and change their own beliefs (21). This model argues that success in modifying health behaviors depends on two basic propositions: First, people must perceive a threat to their health (22). This includes perceived susceptibility, i.e., the belief that they are susceptible to a serious health condition, and perceived severity, which involves the belief that this condition will have serious consequences for their life (9). This perception of threat is important in motivating people to take preventive action. Second, people must perceive that the benefits of taking specific actions to improve their health outweigh any perceived barriers or costs (9). This involves a perception of self-efficacy, where people believe that they have the ability and resources to implement the desired changes in their health behaviors (23). Understanding these benefits and barriers is essential for people to adopt healthy behaviors and maintain these changes over time (22). In the specific case of university students, the model has been shown to be effective in increasing beliefs about healthy lifestyles and developing changes related to healthy eating, regular physical activity, and other health-related behaviors (21, 23, 24).

#### 1.2.1 Beliefs about a healthy lifestyle and life satisfaction

Most research focusses on the relationship between lifestyle practices and life satisfaction, rather than the beliefs of the participants about these practices (6, 25). Furthermore, these studies tend to be specific because they are conducted primarily with health science students rather than the general university population. For example, Senmar et al. (7) found that medical students who maintained an unhealthy lifestyle reported low levels of life satisfaction. Similarly, the study by Badura-Brzoza et al. (25) conducted in medical students reported a positive relationship between life satisfaction and health-promoting behaviors. Likewise, the findings of a study among medical science students in Poland showed that lifestyle is positively related to life satisfaction (6). However, not all studies find these associations. For example, research conducted among U.S. university students found no significant correlations between health-related components and scores on the life satisfaction questionnaire used (26). These findings highlight the need to explore more broadly how lifestyle beliefs affect life satisfaction in a broader and more diverse university population, that is, not just health science students.

### 1.3 Religiosity

Religiosity refers to a person’s devotion and spiritual practices and can have a significant impact on several areas of life, including mental health, life satisfaction, and overall well-being (27, 28). However, as a multidimensional construct, religiosity is related to health outcomes in several dimensions. For example, several dimensions have been identified in the literature, including Organizational Religious Activity (ORA), Non-Organizational Religious Activity (NORA), and Intrinsic Religiosity (IR) (29). ORA, which includes participation in formal religious services and activities, provides a social support network and a sense of belonging. NORA, which includes private practices such as prayer and meditation, provides a personal space for reflection and spiritual connection. IR, on the other hand, involves the internalization of faith and the integration of religious beliefs into all aspects of life (30). In addition, extrinsic religiosity (ER) is another dimension that measures the use of religion for social and personal benefits, such as status and acceptance within a religious community (31). ER can help people establish and maintain useful social networks, although its impact on life satisfaction may vary depending on the depth and sincerity of the underlying religious beliefs (32). These dimensions provide a comprehensive framework to understand how religiosity can affect health and play an important role in promoting the well-being and holistic health of individuals (33).

#### 1.3.1 Religiosity and life satisfaction

Both in the university context and in the general population, several studies have demonstrated that religious individuals tend to enjoy better mental health and lead happier, more fulfilling lives (34–36). In particular, religiosity has been associated with lower levels of academic stress, anxiety, and depression among university students (35, 37). Participation in religious activities often provides a robust social support system and community network, which helps students cope more effectively with academic stress (35). Additionally, religiosity has been linked to a lower incidence of risk behaviors, such as substance abuse, and a greater ability to manage emotional and psychological challenges, thereby reducing levels of anxiety and depression (10).

Furthermore, religious students tend to have greater satisfaction with life and their academic career, which can be attributed to the values and principles instilled in their spiritual beliefs, which provide them with a sense of purpose and direction (38, 39). For example, Aftab et al. (35) reported that unorganized religious activity and intrinsic religiosity were positively associated with life satisfaction among pharmacy students. Similarly, a study of Turkish university students found a significant association between religiosity and happiness after controlling for confounding variables such as gender (36). Using the Individual Religion Inventory, Akbayram et al. (40) also found an association between religion and life satisfaction among medical students in Turkey. These findings underscore the importance of religiosity as a potentially valuable resource to improve the quality of life of students.

However, the findings of a positive association between religiosity and life satisfaction and mental health are not universal. Some studies suggest that religious participation may be associated with poorer mental health and well-being (41), and not necessarily with higher levels of life satisfaction in the general population (28). A study of U.S. adults found that “belief in God” is not necessarily associated with mental health benefits and may be associated with psychiatric symptoms such as social anxiety, paranoia, obsessive thoughts, and compulsions (27). In the university context, a study conducted in Trinidad found that religiosity was significantly and positively associated with life satisfaction in general; however, in some specific demographic groups, such as women, Christians, religiously affiliated and older adult, religiosity was not associated with higher life satisfaction (38). This suggests that the influence of religiosity on life satisfaction may vary according to cultural and demographic context. Similarly, Dankulincova Veselska et al. (42) found no relationship between religiosity and life satisfaction in a group of adolescents. Therefore, according to these findings, religiosity is a complex construct and its influence on life satisfaction can vary among young people.

While the influence of religiosity on life satisfaction may vary across cultural and demographic contexts, it remains a potentially valuable resource that can complement healthy lifestyle beliefs, thereby promoting greater overall well-being. Consequently, both religiosity and lifestyle beliefs should be regarded as key components in efforts to enhance life satisfaction among university students.

### 1.4 Influence of sociodemographic factors

In addition to lifestyle beliefs and religiosity, several sociodemographic factors may also play an important role in shaping life satisfaction among university students (43–46). For example, empirical studies have shown that age can influence life satisfaction; in fact, one study reported that as students’ age increases, their life satisfaction levels tend to decrease (47). Similarly, students from rural backgrounds might face more significant challenges adapting to urban university environments, which could impact their overall well-being and life satisfaction (45). This situation may be similar for international students; for example, the pressure of adapting to a new country and culture, combined with the academic burden, can be an overwhelming experience that affects life satisfaction (44, 47).

On the other hand, health science students may experience greater life satisfaction compared to students in other fields, possibly due to their focus on wellness and quality of life (48, 49). Moreover, marital status is another factor that can influence life satisfaction, as demonstrated by a study conducted on a large sample of 1,031 young adults (50). It is possible that married students or those in stable relationships might report higher life satisfaction due to emotional support from their partners. However, a study by Diener et al. found that individuals who have never been married report higher subjective well-being than those who are divorced, separated, or widowed (51). BMI, in particular, is another important factor, as people with higher BMI levels might experience body dissatisfaction or health concerns, influencing both their satisfaction with life and their beliefs about maintaining a healthy lifestyle (52). These sociodemographic characteristics not only affect life satisfaction but may also moderate the relationship between religiosity, lifestyle beliefs, and overall well-being.

### 1.5 The current study

This study aims to explore how lifestyle beliefs and religion correlate with life satisfaction in the university context. Given the potential influence of sociodemographic factors and the diverse nature of university populations worldwide, this study will also examine how these aspects relate to life satisfaction. Particularly, lifestyle beliefs and religiosity are critical from a clinical and psychosocial perspective, especially considering the emotional and academic challenges faced by university students (5, 10). Universities, as educational institutions, aim to educate everyone regardless of their ethnic origin, and understanding how these factors influence well-being is essential. University students, who are in a transition phase and face many emotional, social, and academic challenges, require a holistic approach that addresses both their religious beliefs and lifestyle habits to promote their overall well-being (6). Assessing how these variables positively correlate with life satisfaction is important to guide health professionals and researchers in understanding and addressing mental health problems that may arise in the university setting.

In addition, in Peru, one of the most religious countries in South America with a significant diversity of faiths (4), it is particularly important for clinicians to understand the relationship between religious behavior and life satisfaction in young people. This understanding may be the key to developing interventions and culturally sensitive strategies that address the specific needs of university students in this multicultural context. In light of the diverse student populations in universities, including foreign and local students, this knowledge can contribute to the formulation of mental health policies and support programs that promote a healthy and balanced university environment that fosters not only academic performance but also the overall well-being of students. Therefore, the objective of this study was to determine the association between sociodemographic aspects, lifestyle beliefs, and religiosity with life satisfaction in Peruvian university students.

## 2 Materials and methods

### 2.1 Study design

A cross-sectional predictive study (53), in which sociodemographic aspects, beliefs about lifestyle, and religiosity were considered predictor variables, while life satisfaction was considered as a criterion variable.

### 2.2 Participants

Data were collected from a group of students at a private university located in the northern region of Peru during the months of March and May 2024. The sample was non-random, which means that participation was voluntary, and students were selected based on their availability and willingness to participate. Specifically, the recruitment process involved collaborating with faculty members and administrative staff from each of the university’s five faculties: Law and Humanities, Business Sciences, Engineering and Architecture, Health Sciences, and Social Sciences. Announcements were made in classrooms, with the help of the teachers, to invite students to participate in the study. The sample size was calculated using Free Statistic Calculators version 4.0 software by Soper (54). Using eight predictors and an expected effect size (*f*^2^ = 0.15), a desired power level = 0.80, and a probability level = 0.05, it was determined that 108 participants would be sufficient to detect effects (55). However, to increase the robustness and validity of the results, and to ensure a more representative sample across the different faculties, the sample size was intentionally expanded. The final sample consisted of 1,258 participants.

Students in the 2024-I semester at the university who gave their informed consent to participate in the study and who were in regular academic status, that is, not on suspension or temporary leave, were included. Students with serious medical conditions or diagnosed psychiatric disorders were excluded, as were those who provided inconsistent or incomplete information on the questionnaires. The study was approved by the Ethics Committee of the Universidad Señor de Sipán. Informed consent was obtained from all participants, whose participation was voluntary and anonymous. The study was carried out in accordance with the principles of the Declaration of Helsinki (56).

### 2.3 Variables

#### 2.3.1 Sociodemographic characteristics

Sociodemographic information was collected using a registration form that included various factors, such as participants’ age, sex (male or female), and place of origin (Peru or abroad). Information was also collected on the students’ place of residence, classified as urban or rural. In addition, the faculties to which the participants belonged were considered, grouped into four categories: Law and Humanities, Business Sciences, Engineering and Architecture, and Health Sciences. The form also inquired about the participants’ marital status, categorized as either single or married. “Finally, data on weight and height were collected to calculate the BMI, which was classified according to the technical guidelines for anthropometric assessment of adults by the Peruvian Ministry of Health as follows (57): underweight (BMI < 18.5), normal weight (BMI ≥ 18.5 to 24.9 kg/m^2^), overweight (BMI 25 to 29.9 kg/m^2^), and obesity (BMI ≥ 30 kg/m^2^).

#### 2.3.2 Beliefs about lifestyles

In the present study, the lifestyle beliefs of the participants were assessed using a 4-item scale (19, 58). The items were designed on a 5-point Likert-type scale (5 = strongly agree and 1 = strongly disagree). An example item on this scale is “*Do you think physical activity affects a person’s general health?*” The scale showed good reliability in our study, with a Cronbach’s alpha of 0.70.

#### 2.3.3 Religiosity

The Santa Clara Short-Scale Strength of Religious Faith Questionnaire (SCSRFQ-SF), which originally had 10 items, was used to assess religiosity (59). The instrument consists of five Likert-type items that ask about the importance of religious belief, regardless of religious affiliation. The response options range from *strongly disagree* = 1 to *strongly agree* = 4 (59). SCSRFQ has been consistently found to be a unidimensional measure with strong reliability and validity between study populations in several countries, including Peru (60, 61). The items were summed to obtain a total religiosity score ranging from 0 to 30. The instrument demonstrates adequate reliability indices (*α* = 0.95; *ω* = 0.94) (60). In this study, Cronbach’s alpha value for the application of SCSRFQ-SF was 0.80, indicating good internal consistency.

#### 2.3.4 Satisfaction with life

The Life Satisfaction Scale (SWLS), translated and validated into Spanish by Atienza et al. and adapted to the Peruvian context by Caycho-Rodríguez et al. (13) was used to assess life satisfaction. This brief scale consists of five items that measure the degree of personal satisfaction with life. The scale uses a five-point Likert format, where responses range from (1) “strongly disagree” to (5) “strongly agree.” An example item of this scale is: *“In most aspects, my life is close to my ideal.”* The scale presents high internal consistency indices (*α* = 0.93 and *ω* = 0.93) (13). In the present study, SWLS showed reliability with an α = 0.68.

### 2.4 Statistical analysis

SPSS version 25 statistical software was used for data processing and analysis. Descriptive analysis was performed using mean and standard deviation. Sociodemographic variables were described using frequency and percentage tables. To assess the significance of differences between groups, Student’s *t*-tests were used for independent samples when the sociodemographic variable had two categories (e.g., sex), and one-way analysis of variance (ANOVA) was used for variables with more than two categories (e.g., age, BMI). Effect sizes for significant differences were calculated using Cohen’s *d* for *t*-tests and Cohen’s *f* for ANOVA. Relationships were analyzed using Pearson’s correlation coefficient. Additionally, multiple linear regression analyses were conducted to determine the relative contribution of lifestyle beliefs, religiosity, and sociodemographic variables in predicting life satisfaction. All relevant sociodemographic variables were included in these models. A significance level of 5% was used for all analyses.

## 3 Results

Table 1 presents the sociodemographic characteristics of the study sample (*N* = 1976). The mean age was 21.86 (SD = 3.40). More than 57% were female (*n* = 725). Approximately 79.9% of the participants reside in urban areas (*n* = 1,005). Approximately 63.7% of the participants were from the faculty of health sciences (*n* = 801). Most of the students were married (94.6%). Almost 40% of the participants were overweight.

**Table 1:** Sociodemographic characteristics of the sample (*N* = 1,258).

	n/M	%/ SD
**Age**	21.86	3.40
**Sex**		
Male	533	42.4
Female	725	57.6
**Provenance**		
Peru	1,171	93.1
Foreign	87	6.9
**Residence**		
Urban	1,005	79.9
Rural	253	20.1
**Faculty**		
Law and Humanities	21	1.6
Business Sciences	249	19.8
Engineering and Architecture	187	14.9
Health Sciences	801	63.7
**Marital status**		
Single	1,190	94.6
Married	68	5.4
**BMI**		
Under	36	2.9
Normal	726	57.7
Overweight	396	31.5
Obesity	99	7.9

BMI, body mass index; M, mean; SD, standard deviation.

Table 2 presents the descriptive results of the study sample. Generally, lifestyle beliefs have a mean (M) of 16.97 (SD = 4.58), religiosity has a mean of 12.49 (SD = 2.19), and life satisfaction has a mean of 22.45 (SD = 6.14). On the other hand, significant differences were observed in beliefs about lifestyles based on sex (*p* = 0.027; *d* = 0.127), residence (*p* = 0.001; *d* = 0.319), faculty (*p* = 0.014; *d* = 0.781), marital status (*p* = 0.017; *d* = 0.141), and age (*p* = 0.012; *f* = 0.084). Regarding religiosity, significant differences were found based on sex (*p* = 0.001; *d* = 0.186), marital status (*p* = 0.001; *d* = 0.415), and age (*p* = 0.001; *f* = 0.204). Regarding life satisfaction, significant differences were found based on origin (*p* = 0.015; *d* = 0.321), residence (*p* = 0.007; *d* = 0.206), faculty (*p* = 0.001; *d* = 0.812), marital status (*p* = 0.001; *d* = 0.191), and age (*p* = 0.001; *f* = 0.111).

**Table 2:** Descriptive and comparative analysis of lifestyle beliefs, religiosity, and life satisfaction across sociodemographic variables.

Characteristics	Lifestyle beliefs		Religiosity		Life satisfaction	
	*p*-value	d/f	*p*-value	d/f	*p*-value	d/f
General (*M* ± SD)	16.97 ± 4.58		12.49 ± 2.19		22.45 ± 6.14	
Sex^a^	0.027	0.127	0.001	0.186	0.071	0.103
Provenance^a^	0.448	0.100	0.069	0.239	0.015	0.321
Residence^a^	0.001	0.319	0.911	0.009	0.007	0.206
Faculty^a^	0.014	0.781	0.124	0.026	0.001	0.812
Marital status^a^	0.017	0.141	0.001	0.415	0.001	0.191
BMI^b^	0.953	0.016	0.843	0.026	0.489	0.045
Age^b^	0.012	0.084	0.001	0.204	0.001	0.111

M, Mean; SD, Standard Deviation.

a Student’s *t*-test.

b One-way ANOVA.

*p, p-value; d, Cohen’s d; f, Cohen’s f; BMI, body mass index.*

Table 3 presents the correlation analysis of the study variables: life satisfaction, lifestyle beliefs, and religiosity. The results indicate a significant positive correlation between life satisfaction and lifestyle beliefs (*r* = 0.262, *p* < 0.01), as well as between life satisfaction and religiosity (*r* = 0.607, *p* < 0.01). In addition, a significant positive correlation is observed between lifestyle beliefs and religiosity (*r* = 0.144, *p* < 0.01). These correlations suggest that as lifestyle beliefs and religiosity increase, life satisfaction also tends to increase in the studied sample.

**Table 3:** Correlation analysis of the study variables.

Variable	Life satisfaction	Lifestyle beliefs	Religiosity
Life satisfaction	1	0.262***	0.607***
Lifestyle beliefs		1	0.144***
Religiosity			1

***p < 0.001; the relationship analysis was performed using Pearson’s correlation coefficient.

Model 2 shows a significant fit both overall (adjusted *R*^2^ = 0.402, *F* = 283.181, *p* < 0.001) and in the individual coefficients (*p* < 0.01). This model indicates that lifestyle beliefs, religiosity, and background are significant predictors of life satisfaction. Among these variables, lifestyle beliefs emerge as the most influential factor, with a standardized coefficient (𝛽 = 0.579) and a highly significant *p*-value (*p* < 0.001). Religiosity also makes a significant contribution (𝛽 = 0.182, *p* < 0.001). Foreign origin has a significant negative effect (𝛽 = −0.066, *p* = 0.003), suggesting that being a foreigner, compared to being Peruvian, is associated with lower life satisfaction. In contrast, belonging to the Faculty of Health Sciences is associated with higher life satisfaction (𝛽 = 0.119, *p* = 0.048). In general, the model explains 40.2% of the variance in life satisfaction (adjusted *R*^2^ = 0.402, *p* < 0.001) (see Table 4).

**Table 4:** Multiple linear regression of factors related to life satisfaction.

	*B*	SE	𝛽	*t*	*p*-value
**Model 1**					
(Constant)	3.253	1.254		2.594	0.010
Lifestyle beliefs	0.776	0.030	0.578	25.958	<0.001***
Religiosity	0.498	0.064	0.177	7.794	<0.001***
Sex	−0.062	0.275	−0.005	−0.225	0.822
Provenance	−1.851	0.628	−0.065	−2.947	0.003
Residence	−0.031	0.367	−0.002	−0.084	0.933
Faculty	0.054	0.027	0.119	1.984	0.048
Marital status	0.046	0.380	0.004	0.122	0.903
BMI	−0.017	0.037	−0.011	−0.474	0.636
Age	0.011	0.015	0.022	0.742	0.458
**Model 2**					
(Constant)	2.953	0.869		3.397	0.001
Lifestyle beliefs	0.778	0.030	0.579	26.288	<0.001***
Religiosity	0.511	0.062	0.182	8.242	<0.001***

Dependent variable: Life satisfaction; B, Non-standardized regression coefficient; SE, Standard error; 𝛽 (Beta), Standardized regression coefficient; *t*, t-statistic; *p*-value, Probability value indicating the level of statistical significance; *p* < 0.001***; *p* < 0.01**; Model 1: adjusted *R*^2^ = 0.401, *F* = 106.020, *p* < 0.001; Model 2: adjusted *R*^2^ = 0.402, *F* = 283.181, *p* < 0.001.

## 4 Discussion

### 4.1 Summary

Life satisfaction is an important indicator of general well-being, especially in the university context where students face a variety of academic, social, and personal challenges (35). During this transitional phase, beliefs and practices related to healthy lifestyles and religiosity, along with sociodemographic factors such as provenance, faculty, and BMI, can significantly influence how students manage their overall well-being (19, 35, 36). In Peru, a country with rich religious and cultural diversity, it is particularly relevant to explore how these factors can influence the life satisfaction of university students. The purpose of this study was to examine the relationship between lifestyle beliefs, religiosity, and life satisfaction in a sample of Peruvian university students. Among the main findings of the present study, we found that (a) Being an international student, compared to a local student, is negatively and significantly associated with life satisfaction; (b) A positive association was observed between belonging to the Faculty of Health Sciences and life satisfaction, compared to other faculties; (c) beliefs about lifestyles are positively and significantly associated with life satisfaction; and (b) religiosity significantly predicts life satisfaction.

### 4.2 Sociodemographic aspects

International students may face additional challenges that negatively affect their overall well-being. These challenges include language barriers, cultural differences, and a lack of established social support networks, which can contribute to feelings of isolation and difficulty in adjusting to the university environment. The pressure of adapting to a new country and culture, along with the academic load, can be overwhelming and reduce their perception of life satisfaction. In the current study, we found that being from a foreign background, compared to being Peruvian, is negatively and significantly associated with life satisfaction. Our findings are consistent with previous studies that have reported international students tend to experience lower levels of subjective well-being compared to their local counterparts, partly due to disconnection from their family support systems and academic expectations that may be more difficult to meet in a foreign environment (44, 47). In addition, foreign students often experience higher levels of stress and anxiety due to acculturation, perceived discrimination, and difficulties in accessing academic and social support resources (62). However, a study conducted on Polish university students reported no significant difference in the life satisfaction of international students compared to local students (6). Nevertheless, it is fundamental for higher education institutions to recognize the unique needs of international students and develop targeted strategies to support their adaptation and well-being.

Another relevant finding of the current study is that being part of the Faculty of Health Sciences is significantly associated with higher life satisfaction. This suggests that the faculty to which students belong plays an important role in their overall well-being and personal satisfaction. Previous studies exploring life satisfaction in the university context often find that students in health-related faculties, due to their focus on wellness and quality of life, tend to report higher levels of life satisfaction (48, 49). Additionally, other research has shown that students in health-related fields, being more aware of factors that contribute to well-being, such as stress management and mental health care, tend to apply this knowledge in their daily lives, which could explain their higher life satisfaction (25).

The higher life satisfaction reported by health sciences students compared to those in other faculties may be attributed to several interrelated factors (43, 63–65). For example, these students are regularly exposed to academic content that emphasizes the importance of physical, mental, and emotional well-being (64). This not only increases their knowledge of healthy practices but also reinforces the adoption of these behaviors in their daily lives. Furthermore, an emphasis on nutrition and healthy eating, regular physical exercise, and stress management can lead to improved self-regulation and greater engagement in activities that promote personal wellness (65). Another relevant factor is that, by being immersed in a discipline focused on improving the health and well-being of others, they are likely to experience greater satisfaction from the sense of purpose and personal fulfillment that comes with helping others (63). This sense of purpose can not only enhance academic motivation but also contribute to greater overall life satisfaction (43).

### 4.3 Beliefs about lifestyle

In the current study, beliefs about lifestyle were found to be positively and significantly associated with life satisfaction among the Peruvian university students evaluated. This suggests that students who have strong, positive beliefs about the importance of leading a healthy lifestyle tend to report higher levels of life satisfaction. This finding underscores the importance of promoting healthy beliefs and practices among students as a strategy to improve their general well-being. It should be mentioned that, in the current study, the assessment of beliefs about lifestyles was based on the influence of several key components on general health, such as regular physical activity, a balanced diet, tobacco use and stress management (19). These components are widely recognized in the literature for their positive effects on physical and mental health.

The findings of our study are consistent with previous studies that have demonstrated a positive association between some of these components of healthy lifestyles and greater subjective well-being. For example, the results of a study of 17 to 30 students from 21 countries found that those who practiced healthy habits such as eating fruit and limiting fat intake, exercising regularly, and not smoking reported greater life satisfaction and a better overall mood (66). Similarly, a study of Chilean university students found that participants who reported eating at home more often, had healthy eating habits, and considered food very important to their well-being had higher levels of life satisfaction (67). Furthermore, in line with our findings, an online survey conducted in Chinese university students showed that lifestyle has a positive relationship with life satisfaction (68). On the other hand, stress management is a component that plays an important role in student life satisfaction. For example, a study in university students found that stress was negatively correlated with life satisfaction (69).

However, the association between lifestyle aspects and life satisfaction among university students is not unanimous. For example, research conducted among U.S. university students found no significant correlations between the health-related components of physical fitness and life satisfaction (26). Regardless, the consistency of these findings with the existing literature suggests that interventions that promote healthy beliefs may have a positive impact on university students’ life satisfaction. By integrating educational programs that emphasize the importance of nutrition, physical activity, stress management, and other healthy behaviors, educational institutions can help students develop behaviors that promote long-term wellness.

### 4.4 Religiosity

Another important finding of the current study is that religiosity significantly predicts life satisfaction. This suggests that students with high levels of religiosity tend to report higher levels of life satisfaction. This finding may be related to several factors intrinsic to religious practice, such as a sense of belonging to a community, social and emotional support (35). These elements can contribute to a greater sense of purpose and general well-being, which is reflected in greater life satisfaction (70). Consistent with our findings, one study has shown that aspects of religiosity that increase social capital and strengthen social bonds, such as attendance at religious services, are positively correlated with life satisfaction (33). Previous studies of college students have found that regular attendance to the church and spiritual practices are associated with lower levels of stress, anxiety, and depression, as well as greater life satisfaction and emotional well-being (10, 34, 35, 37). Furthermore, Aftab et al. (35) found that unorganized religious activity and intrinsic religiosity were positively associated with life satisfaction in university students. Similarly, Akbayram et al. (40) found an association between religion and life satisfaction in medical students.

However, our findings differ from some studies suggesting that religiosity or belief in God is not associated with life satisfaction (27, 28, 41, 42). For example, Habib et al. (38), found that while there was an association between religiosity and life satisfaction among university students, there was no significant association among some specific demographic groups, such as women, Christians, religiously affiliated and the older adult. Furthermore, studies such as those of Silton et al. (27) have shown that “belief in God” is not necessarily associated with mental health benefits and can correlate with psychiatric symptoms such as social anxiety, paranoia, obsessive thoughts, and compulsions. These results underscore the need for a careful interpretation of the relationship between religiosity and well-being, recognizing that the effects may vary significantly depending on the context and demographic characteristics of the population studied. In any case, the positive relationship between religiosity and life satisfaction observed in our study underscores the importance of considering spirituality and religion as relevant factors in promoting well-being among university students. Fostering an environment that respects and supports religious diversity can contribute significantly to improving the quality of life and general well-being of the student population.

### 4.5 Public health implications

These findings have important implications for public health policy and university wellness programs. For example, it is important for universities to implement support programs that promote social integration and provide access to wellness services for international students, improving their quality of life and reducing stress factors. On the other hand, given that health sciences students exhibit higher levels of life satisfaction, it is important to promote health education across all faculties, not just those related to health sciences, in order to improve the overall well-being of the student population.

Additionally, because beliefs about healthy lifestyles are so closely related to life satisfaction, it is important for universities to provide resources and support to help students adopt and maintain these habits. These can range from workshops and seminars on healthy eating and nutrition, regular physical activity, the negative effects of risky behaviors such as alcohol, tobacco, and general substance abuse, to counseling and psychological support services to help students manage stress and maintain a healthy balance between their studies and their personal lives.

On the other hand, in an environment where students face multiple academic, social, and personal challenges, organized religious activities, non-organized religious activities, and intrinsic religiosity may serve as valuable resources for coping with stress and promoting well-being. Universities could consider implementing programs and activities that support students’ religious and spiritual practices, such as discussion groups, meditation workshops, and prayer rooms. Furthermore, university wellness and counseling services, which include physical and mental health, academic support, personal and social development, and promotion of healthy lifestyles, could integrate the spiritual and religious dimension into their interventions to provide comprehensive support to students.

## 5 Limitations and future considerations

Although this study has provided important findings on the relationship between lifestyle beliefs, religiosity, and life satisfaction among Peruvian university students, it is crucial to consider several limitations when interpreting the results. First, this is a cross-sectional study, which means that causal relationships cannot be established between the variables evaluated. Data collected at a single point in time do not allow us to determine whether belief and religiosity influence life satisfaction or vice versa. Second, data collection was conducted at a single university, which can limit the generalizability of the findings to other educational institutions and cultural contexts. In addition, sampling was non-probability by convenience, which may introduce bias into the sample and affect the representativeness of the results. It is possible that the students who chose to participate have different characteristics from those who did not, which could influence the results obtained.

Another significant limitation is the use of self-report scales to assess the variables of religiosity, life satisfaction, and beliefs about lifestyle. Although these scales are validated and commonly used tools in research, they rely on the perception and honesty of participants in responding, which can introduce response bias and affect the accuracy of the data. Furthermore, the study did not consider other potentially influential variables, such as socioeconomic status, family history of mental and physical health, and other environmental factors that could have moderated or mediated the relationships found. For future research, it would be beneficial to conduct longitudinal studies to observe changes over time and establish causal relationships. It would also be valuable to expand the sample to include students from different universities and regions of the country, which would increase the generalizability of the results. Additionally, considering the use of random sampling methods and the inclusion of additional variables could provide a more complete and nuanced view of the factors that influence life satisfaction among university students.

## 6 Conclusion

This cross-sectional study found that both beliefs about lifestyles and religiosity were significant predictors of life satisfaction among Peruvian university students. Furthermore, the study revealed that being an international student is negatively associated with life satisfaction. On the other hand, students from health sciences were found to have higher life satisfaction. Therefore, this study highlights the need for universities and health professionals to consider promoting healthy lifestyles, supporting religious practices, and providing targeted support for international students as important strategies for improving student life satisfaction.

## Data Availability

The raw data supporting the conclusions of this article will be made available by the authors, without undue reservation.
